# Excessive *All-Trans* Retinoic Acid Inhibits Cell Proliferation Through Upregulated MicroRNA-4680-3p in Cultured Human Palate Cells

**DOI:** 10.3389/fcell.2021.618876

**Published:** 2021-01-28

**Authors:** Hiroki Yoshioka, Sai Shankar Ramakrishnan, Junbo Shim, Akiko Suzuki, Junichi Iwata

**Affiliations:** ^1^Department of Diagnostic and Biomedical Sciences, School of Dentistry, The University of Texas Health Science Center at Houston, Houston, TX, United States; ^2^Center for Craniofacial Research, The University of Texas Health Science Center at Houston, Houston, TX, United States; ^3^MD Anderson Cancer Center UTHealth Graduate School of Biomedical Sciences, Houston, TX, United States

**Keywords:** all-trans retinoic acid (all-trans RA), cleft palate (CP), microRNA (miR), cell proliferation, environmental factor

## Abstract

Cleft palate is the second most common congenital birth defect, and both environmental and genetic factors are involved in the etiology of the disease. However, it remains largely unknown how environmental factors affect palate development. Our previous studies show that several microRNAs (miRs) suppress the expression of genes involved in cleft palate. Here we show that *miR-4680-3p* plays a crucial role in cleft palate pathogenesis. We found that *all-trans* retinoic acid (*at*RA) specifically induces *miR-4680-3p* in cultured human embryonic palatal mesenchymal (HEPM) cells. Overexpression of *miR-4680-3p* inhibited cell proliferation in a dose-dependent manner through the suppression of expression of *ERBB2* and *JADE1*, which are known cleft palate-related genes. Importantly, a *miR-4680-3p*-specific inhibitor normalized cell proliferation and altered expression of *ERBB2* and *JADE1* in cells treated with *at*RA. Taken together, our results suggest that upregulation of *miR-4680-3p* induced by *at*RA may cause cleft palate through suppression of *ERBB2* and *JADE1*. Thus, miRs may be potential targets for the prevention and diagnosis of cleft palate.

## Introduction

The prevalence of cleft lip with or without cleft palate (CL/P) is ~1 in 700 live births worldwide (Ferguson, 1988), and affected individuals require surgical repairs, speech therapy, and dental treatments (Ferguson, 1988). Palate development (which consists of the growth, elevation, and fusion of the palatal shelves) starts at 6–8 weeks of gestation in humans; any failure in these process results in cleft palate (Habib, 1978; Mossey et al., 2009). The etiology of cleft palate is complex and involves environmental and genetic factors and their interactions (Dhulipala et al., 2006; Havasi et al., 2013; Buser and Pohl, 2015; Liu et al., 2018). Maternal use and exposure to tobacco, alcohol, drugs (e.g., retinoic acid and dexamethasone), and chemicals (e.g., dioxin and heavy metals), as well as mutations in genes related to the degradation/metabolism/release of these teratogens, are considered to be risk factors (Prescott et al., 2002; Chevrier et al., 2005; Ramirez et al., 2007). An increasing number of studies suggest that microRNAs (miRs), which are endogenous small non-coding RNAs (~22 nucleotides long) that negatively regulate the expression of their target genes (Bartel, 2004; Obernosterer et al., 2006), play important roles in normal palate development and CL/P in humans and mice (Karsy et al., 2010; Shin et al., 2012; Seelan et al., 2014; Chung et al., 2016; Schoen et al., 2017, 2018; Wang et al., 2017; Mukhopadhyay et al., 2019); however, it remains elusive how and which miRs are crucial roles in CL/P. Our recent studies show that overexpression of either *miR-140-5p, miR-133b, miR-374a-5p, miR-381-3p*, or *miR-4680-3p* suppresses cell proliferation in cultured human embryonic palatal mesenchymal (HEPM) cells (Li et al., 2019; Suzuki et al., 2019), suggesting that these miRs may be involved in the pathogenesis of cleft palate.

*All-trans* retinoic acid (*at*RA), a derivative of vitamin A, is the most active retinoid that plays important roles in a variety of biological processes, including cell proliferation, differentiation, and extracellular matrix production (Wang and Kirsch, 2002; Lai et al., 2003; Rhinn and Dolle, 2012; Cunningham and Duester, 2015). While *at*RA is widely used in the treatments of skin diseases and cancers (Karsy et al., 2010; Siddikuzzaman and Berlin Grace, 2011; Mihaly et al., 2012), excessive intake of *at*RA, a known teratogen, disrupts embryogenesis, causing birth defects (Abbott et al., 1989; Ross et al., 2000; Roberts, 2020) including cleft palate (Abbott et al., 1989; Ross et al., 2000; Wang and Kirsch, 2002; Lai et al., 2003; Yao et al., 2011; Havasi et al., 2013; Hu et al., 2013; Hou et al., 2019; Roberts, 2020). Recent studies show that *at*RA alters the expression of miRs in human cancer cell lines (Liu et al., 2018, 2019). In this study, we will determine whether and how *at*RA can alter miR expression, which suppresses the expression of genes related to CL/P.

## Methods

### Cell Culture

HEPM cells were obtained from American Type Culture Collection (CRL-1486; ATCC) and maintained under Minimum Essential Medium Eagle-alpha modification (αMEM), supplemented with 10% fetal bovine serum (FBS), penicillin/streptomycin, and L-glutamine, at 37°C in a humidified atmosphere with 5% CO_2_.

### Cell Proliferation Assay

The cells were plated onto 96-well cell culture plates at a density of 5,000/well and treated with *at*RA (R2625, Sigma-Aldrich) at various concentrations (0, 1, 3, 10, and 30 μM), small interfering RNA (siRNA) for either *ERBB2* (#103546; Thermo Fisher Scientific) or *JADE1* (#109590; Thermo Fisher Scientific), or a negative control (#AM4611; Thermo Fisher Scientific), at 3 pmol in 0.3 μL of transfection reagent (TransIT-X2 system; Mirus Bio LLC) in 0.1 mL αMEM per well for 24, 48, or 72 h. Cell proliferation was measured using Cell Counting Kit 8 (Dojindo Molecular Technologies, Inc.) (*n* = 6 per group).

### Bromodeoxyuridine (BrdU) Incorporation Assay

The cells were plated onto 35-mm dishes at a density of 25,000/dish and treated with 30 μM *at*RA, or vehicle (dimethyl sulfoxide). After 72 h, the cells were incubated with BrdU for 1 h. Incorporated BrdU was stained with a rat monoclonal antibody against BrdU (ab6326; Abcam, 1:1,000), as previously described (Suzuki et al., 2018a). A total of nine fields, which were randomly selected from three independent experiments, was used for the quantification of BrdU-positive cells.

### Terminal 2′-Deoxyuridine, 5′-Triphosphate (dUTP) Nick-End Labeling (TUNEL) Staining

The cells were plated onto 35-mm dishes at a density of 25,000/dish and treated with 30 μM *at*RA or vehicle for 72 h. The Click-iT Plus TUNEL Assay with Alexa 594 (C10618, Molecular Probes) was used to detect apoptotic cells, as previously described (Suzuki et al., 2018b). A total of four fields, which were randomly selected from two independent experiments, was used for the quantification of TUNEL-positive cells.

### Quantitative RT-PCR

The cells were plated onto 60-mm dishes at a density of 50,000/dish and treated with 30 μM *at*RA or vehicle. After 24 or 72 h, total RNA isolated from HEPM cells (*n* = 6 per group) was extracted with the QIAshredder and miRNeasy Mini Kit (QIAGEN), according to the manufacturer's instructions. Total RNA (1 μg) from each sample was reverse-transcribed using iScript Reverse Transcription Supermix for quantitative RT-PCR (Bio-Rad), and then the cDNA was amplified with iTaq Universal SYBR Green Supermix (Bio-Rad) using a CFX96 Touch Real-Time PCR Detection system (Bio-Rad). The following PCR primers were used: *ERBB2* (NM_004448) sense, 5′-CATTGGGACCGGAGAAACCA-3′, and antisense, 5′-CGCAGCTTCATGTCTGTGC-3′; *JADE1* (NM_199320) sense, 5′-AAACGCCAGACCGAGAGTG-3′, and antisense, 5′-AGTTGACAGGCTGCCATTGT-3′; *MTHFD1* (NM_005956) sense, 5′-TCCAGTAGTAGTGGCCGTGA-3′, and antisense, 5′-GCTTTGTGTTGAGCTTCGGG-3′; *WNT5A* (NM_003392) sense, 5′-AAGCAGACGTTTCGGCTACA-3′, and antisense, 5′-GCGCCCAATACGACCAAATC-3′; and *GAPDH* (NM_002046) sense, 5′-GACAGTCAGCCGCATCTTCT-3′, and antisense, 5′-GCGCCCAATACGACCAAATC-3′. The amount of each quantified target mRNA was normalized by *GAPDH*. miR expression was measured using the Taqman Fast Advanced Master Mix and Taqman Advanced miR cDNA Synthesis Kit (Thermo Fisher Scientific), according to the manufacturer's instructions. Probes for miR-140-5p (477909_mir), miR-133b (480871_mir), miR-374a-5p (478238_mir), miR381-3p (477816_mir), miR-4680-3p (480701_mir), and miR-26a-5p (477995_mir) were obtained from Thermo Fisher Scientific.

### Immunoblotting

The cells were plated onto 60-mm dishes at a density of 50,000/dish and treated with either *at*RA for 72 h or siRNA for 48 h, as described above. Treated cells were lysed with RIPA buffer (Cell Signaling Technology) containing a protease inhibitor cocktail (Roche). The cells were harvested and centrifuged at 21,130 × *g* for 10 min at 4°C, and the supernatant of each sample was collected and protein level was determined using the BCA protein kit (Pierce). Protein samples were applied to Mini-PROTEAN TGX Gels (Bio-Rad) and transferred to a polyvinylidene difluoride (PVDF) membrane. Mouse monoclonal antibodies against ERBB2 (MA5-13675, Thermo Fisher Scientific, 1:2,000), JADE1 (MAB6275, R&D, 1:2,000), CDKN1B (3,698, Cell Signaling Technology, 1:1,000), and GAPDH (MAB374, Millipore, 1:6,000), rabbit monoclonal antibodies against CCND1 (2,978, Cell Signaling Technology, 1:1,000), phosphorylated ERK1/2 (4,370, Cell Signaling Technology, 1:1,000), ERK1/2 (4,695, Cell Signaling Technology, 1:1,000), phosphorylated mTOR (5,536, Cell Signaling Technology, 1:1,000), and mTOR (2,983, Cell Signaling Technology, 1:1,000), and a rabbit polyclonal antibody against cleaved caspase 3 (9,661, Cell Signaling Technology, 1:1,000), were used for immunoblotting. Peroxidase-conjugated anti-mouse IgG (7,076, Cell Signaling Technology, 1:100,000) and anti-rabbit IgG (7,074, Cell Signaling Technology, 1:100,000) were used as secondary antibodies. All immunoblotting experiments were performed at least two times to validate the results.

### Immunofluorescence Analysis

The cells were plated onto 35-mm glass-bottom dishes at a density of 10,000/dish and treated with 30 μM *at*RA or vehicle control for 72 h. The immunofluorescence analysis was performed as previously described (Suzuki et al., 2018a), using mouse monoclonal antibodies against ERBB2 (MA5-13675, Thermo Fisher Scientific, 1:200) and JADE1 (MAB6275, R&D, 1:200). Images were taken with a confocal microscope (Ti-E, Nikon).

### Knockdown and Overexpression of *ERBB2* and *JADE1*

For the siRNA experiments, the cells were plated onto 35-mm dishes at a density of 20,000/dish. When the cells reached 70% confluency, they were treated with siRNA for either *ERBB2, JADE1*, or negative control, at 3 pmol in 6 μL of transfection reagent (TransIT-X2 system) in 2 mL of αMEM per dish. Total RNA was isolated after 24 h, and total protein was collected after 48 h. For the overexpression of *ERBB2* and *JADE1*, HEPM cells were plated onto 35-mm dishes at a density of 20,000/dish. When the cells reached 70% confluency, they were treated with plasmid DNA for either *ERBB2* [pcDNA3-HER2 (provided by Dr. Mien-Chie Hung through addgene, 16,257)], *JADE1* [pCMV-SPORT6-PHF17 (ABIN3826934; genomics-online.com)], or negative controls [pcDNA3.1-RGS-6xHis (provided by Dr. Adam Antebi through addgene, 52,534) or pCMV-HA (provided by Dr. Christopher A Walsh through addgene, 32,530)], at 500 ng in 6 μL of transfection reagent (TransIT-X2 system) in 2 mL of αMEM per dish. Total RNA was isolated after 24 h.

### Rescue Experiments

The cells were plated onto 60-mm dishes at a density of 250,000/well and treated with 30 μM *at*RA or vehicle control. After 24 h, the cells were transfected with either miR-4680-3p inhibitor (3 pmol) or control miR inhibitor (3 pmol; mirVana, Thermo Fisher Scientific), and either *ERBB2, JADE1*, or control overexpression vector, using the TransIT-X2 system (Mirus Bio LLC), according to the manufacturer's protocol (at 1 μg in 12 μL of transfection reagent in 4 mL of αMEM per dish). The cells were harvested 24 h after transfection and used for further experiments.

### Statistical Analysis

All experiments were performed independently at least two times. The statistical significance of the differences between two groups was evaluated using a two-tailed Student *t*-test. Multiple comparisons were made by one-way analysis of variance with the *post-hoc* Tukey–Kramer's test. A *p* < 0.05 was considered to be statistically significant. Data are represented as mean ± standard deviation in the graphs.

## Results

### *at*RA Inhibits Cell Proliferation in a Dose-Dependent Manner in HEPM Cells

**Figure 1:**
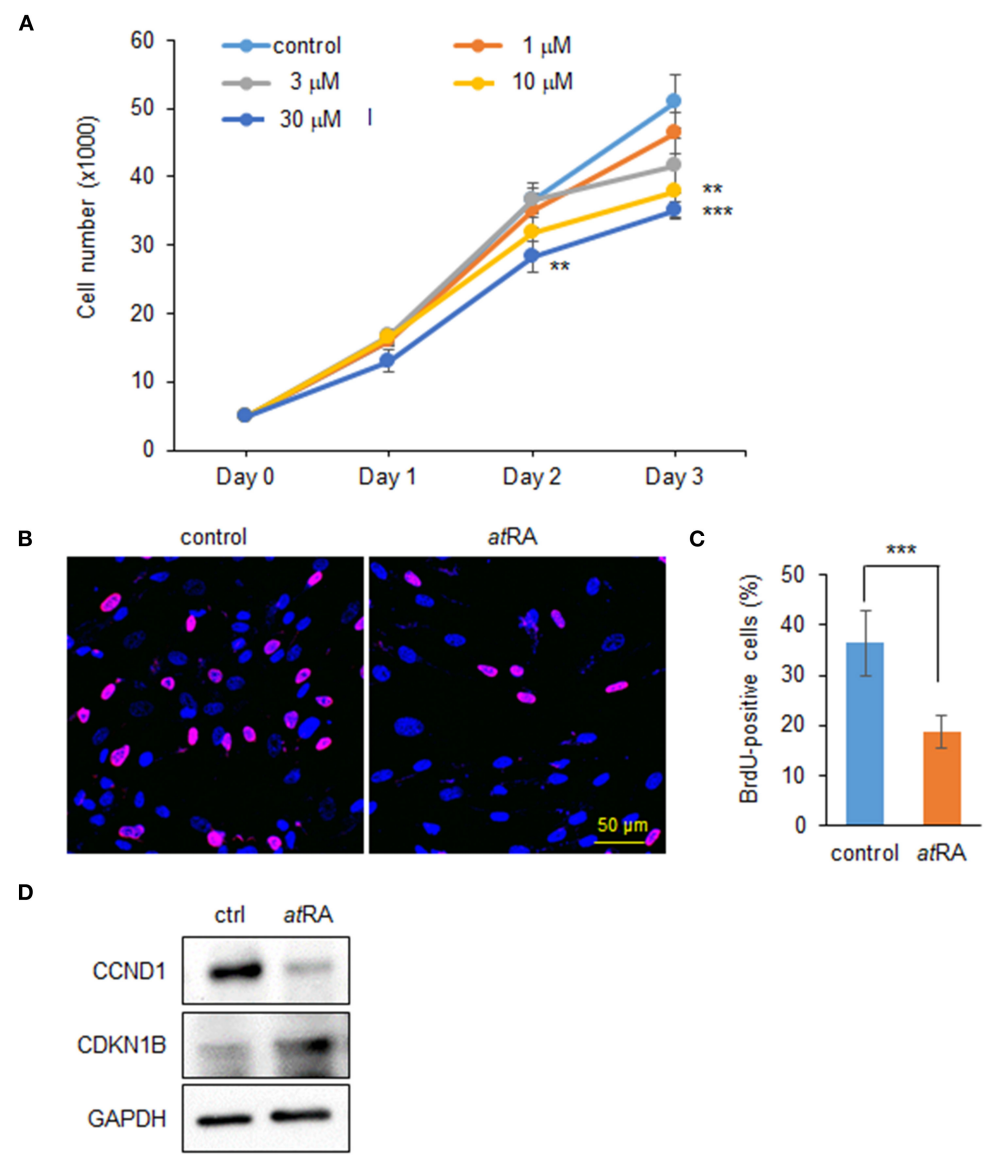
Influence of *at*RA treatment on proliferation of HEPM cells. **(A)** Cell proliferation assays in HEPM cells treated with various concentration of *at*RA for 24, 48, and 72 h. **p < 0.01, ***p < 0.001. Each treatment group was compared with a control vehicle group at each indicated day. **(B)** BrdU staining (red) in HEPM cells after treatment with 30 μM *at*RA for 72 h. Nuclei were counterstained with DAPI (blue). Scale bar, 50 μm. **(C)** Graph shows the quantification of BrdU-positive cells. ***p < 0.001. **(D)** Immunoblotting for CCND1, CDKN1B, and GAPDH in HEPM cells treated with 30 μM *at*RA for 72 h. Representative images from two independent experiments are shown.

To determine the dose-dependent effects of *at*RA on cell proliferation in HEPM cells, we performed cell proliferation assays using HEPM cells treated with *at*RA at various concentrations (0, 1, 3, 10, and 30 μM). We found that cell proliferation activity was decreased by *at*RA treatment in a dose-dependent manner (Figure 1A). BrdU incorporation assays confirmed that cell proliferation was significantly decreased in cells treated with 30 μM *at*RA (Figures 1B,C). While excessive *at*RA is known to induce apoptosis in several tissues and cells (Okano et al., 2007; Mercader et al., 2008; Nelson et al., 2019; Quan et al., 2019), *at*RA failed to induce apoptosis in HEPM cells at 30 μM (Supplementary Figure 1). Previous studies show that *at*RA inhibits cell proliferation through downregulation of cyclin D1 (CCND1) and upregulation of cyclin-dependent kinase inhibitor 1B (CDKN1B; a.k.a. p27, KIP1) in HEPM cells (Dong et al., 2017) as well as in MCF-7 cells, a human breast cancer cell line (Teixeira and Pratt, 1997). We therefore evaluated the expression of CCND1 and CDKN1B by immunoblotting and confirmed that CCND1 expression was downregulated, and CDKN1B expression was upregulated, with *at*RA treatment (Figure 1D).

### *at*RA Suppresses ERBB2 and JADE1 Expression Through Upregulation of *miR-4680-3p* in HEPM Cells

**Figure 2:**
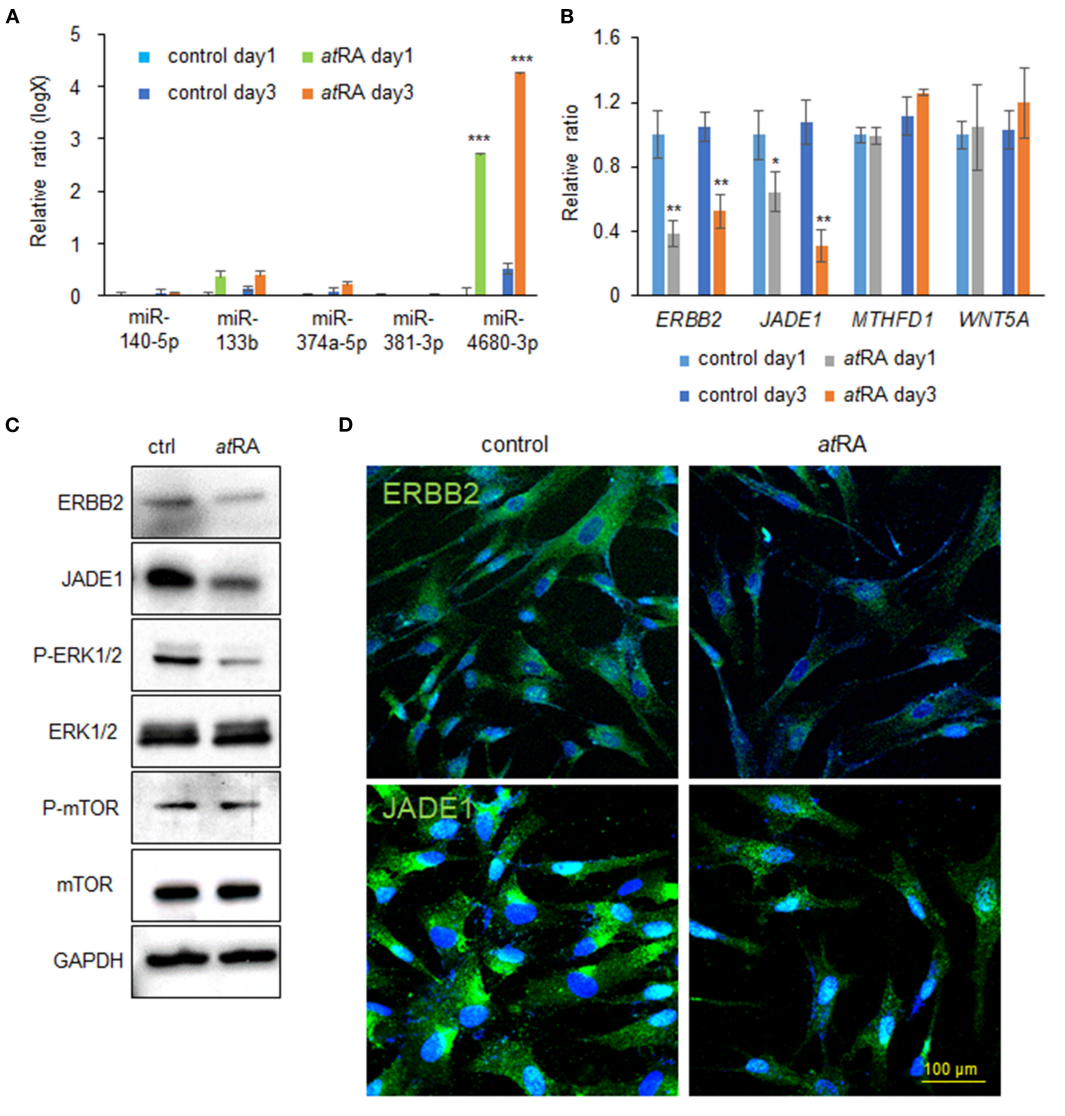
*at*RA induces *miR-4680-3p* expression in HEPM cells. **(A)** Quantitative RT-PCR for the indicated miRs after treatment of HEPM cells with *at*RA for 1 or 3 days. ***p < 0.001. **(B)** Quantitative RT-PCR for the indicated genes after treatment of HEPM cells with *at*RA for 1 or 3 days. *p < 0.05, **p < 0.01. Each treatment group was compared with a control vehicle group at each indicated day. **(C)** Immunoblotting for ERBB2, JADE1, phosphorylated ERK1/2 (P-ERK1/2), ERK1/2, phosphorylated mTOR (P-mTOR), mTOR, and GAPDH in HEPM cells treated with 30 μM *at*RA for 72 h. Representative images from two independent experiments are shown. **(D)** Immunocytochemical analysis of ERBB2 and JADE1 (green) in HEPM cells treated with 30 μM *at*RA for 72 h. Nuclei were counterstained with DAPI (blue).

Our previous studies showed that overexpression of either *miR-140-5p, miR-133b, miR-374a-5p, miR-381-3p*, or *miR-4680-3p* inhibits proliferation of HEPM cells through the suppression of genes that are crucial for palate development (Li et al., 2019; Suzuki et al., 2019). We therefore hypothesized that *at*RA induces the expression of these miRs. To test that hypothesis we analyzed the expression of these miRs after treatment of HEPM cells with *at*RA for 24 and 72 h, and found that expression of *miR-4680-3p*, but not *miR-140-5p, miR-133b, miR-374a-5p*, or *miR-381-3p*, was specifically and significantly upregulated with *at*RA treatment (Figure 2A). Next, to identify target genes suppressed by a miR-4680-3p mimic, we performed quantitative RT-PCR (qRT-PCR) analyses for the target genes (*ERBB2, JADE1, MTHFD1*, and *WNT5A*), which were predicted through bioinformatic analysis (Suzuki et al., 2019). We found that expression of *ERBB2* [a.k.a. *HER2*, a member of the epidermal growth factor receptor (EGFR) family of transmembrane tyrosine kinase-type receptors (Schechter et al., 1984)] and *JADE1* [a.k.a. *PHF17*, a member of the extended plant homeodomain (PHD) finger protein subfamily (Tzouanacou et al., 2003)] was significantly downregulated in HEPM cells treated with *at*RA for 24 and 72 h (Figure 2B). This suppression of ERBB2 and JADE1 in *at*RA-treated cells was confirmed with immunoblotting after 72 h of *at*RA treatment (Figure 2C).

Previous studies showed that ERBB2 stimulates several intracellular pathways such as MAPK/ERK and PI3K/AKT/mTOR (Yarden and Pines, 2012; Croessmann et al., 2019). For this reason, we analyzed the ERK1/2 and mTOR pathways in cells treated with *at*RA and found that ERK1/2 phosphorylation was downregulated, while mTOR phosphorylation was not altered, with *at*RA treatment (Figure 2C). ERBB2 was detected in the plasma membrane and cytosol in controls, as previously reported (Chung et al., 2016); by contrast, its expression was significantly decreased in *at*RA-treated cells (Figure 2D). JADE1 was detected in the nucleus and cytosol in controls, as previously reported (Panchenko et al., 2004; Havasi et al., 2013; Siriwardana et al., 2015); however, JADE1 expression was significantly decreased in *at*RA-treated HEPM cells (Figure 2D). Thus, our results indicate that *at*RA induces *miR-4680-3p* expression, leading to the suppression of ERBB2 and JADE1 via ERK1/2 signaling in HEPM cells.

**Figure 3:**
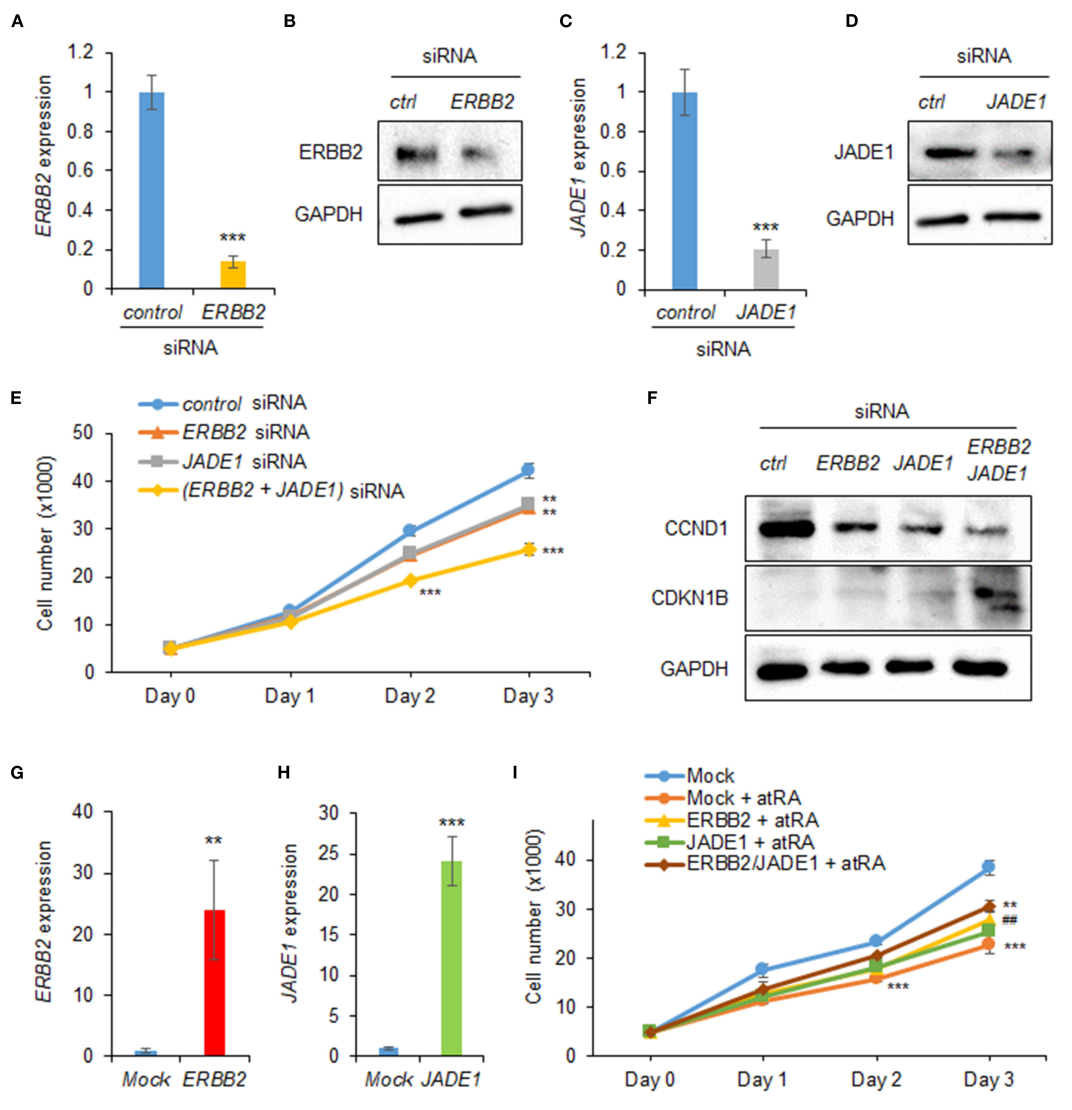
*ERBB2* and *JADE1* knockdown inhibits cell proliferation in HEPM cells. **(A)** Quantitative RT-PCR for *ERBB2* after treatment with *ERBB2* siRNA for 24 h in HEPM cells. ***p < 0.001. **(B)** Immunoblotting of ERBB2 and GAPDH in HEPM cells treated with an *ERBB2* siRNA for 48 h. Representative images from two independent experiments are shown. **(C)** Quantitative RT-PCR for *JADE1* after treatment with *JADE1* siRNA for 24 h in HEPM cells. ***p < 0.001. **(D)** Immunoblotting of JADE1 and GAPDH in HEPM cells treated with *JADE1* siRNA for 48 h. Representative images from two independent experiments are shown. **(E)** Cell proliferation assays in HEPM cells treated with an *ERBB2* or *JADE1* siRNA for 24, 48, or 72 h. **p < 0.01, ***p < 0.001. Each treatment group was compared with a control siRNA group at each indicated day. **(F)** Immunoblotting of CCND1, CDKN1B, and GAPDH in HEPM cells treated with siRNA for *ERBB2* or *JADE1* for 48 h. Representative images from two independent experiments are shown. **(G)** *ERBB2* expression following overexpression in HEPM cells. **p < 0.01. **(H)** *JADE1* expression following overexpression in HEPM cells. ***p < 0.001. **(I)** Cell proliferation assays in HEPM cells overexpressing *ERBB2* and/or *JADE1* for 24, 48, or 72 h. **p < 0.01, ***p < 0.001. Each treatment group was compared with control vector (mock) group at each indicated day. ^##^*p* < 0.01 vs. mock + *at*RA at day 3.

Next, to evaluate the effect of expression of *ERBB2* and *JADE1* on cell proliferation, we treated HEPM cells with siRNAs for *ERBB2* and *JADE1*. We confirmed that siRNA knockdown of either *ERBB2* or *JADE1* suppressed their expression at the mRNA and protein levels (Figures 3A–D). Under these conditions, cell proliferation was significantly suppressed by either *ERBB2* or *JADE1* siRNA knockdown. In addition, additional suppression was observed with a combination of *ERBB2* and *JADE1* siRNAs (Figure 3E). Furthermore, we confirmed that knockdown of *ERBB2* and *JADE1* in HEPM cells resulted in downregulated CCND1 and upregulated CDKN1B (Figure 3F). To evaluate the functional significance of ERBB2 and JADE1, we conducted rescue experiments by overexpressing *ERBB2* and *JADE1* in cells treated with *at*RA. We first confirmed that expression of *ERBB2* and *JADE1* was significantly upregulated following overexpression of these genes (Figures 3G,H). Under these conditions, we found that overexpression of *ERBB2* and *JADE1* partially rescued the cell proliferation inhibited by *at*RA (Figure 3I). Taken together, our results indicate that *at*RA inhibits cell proliferation through dysregulation of the ERBB2/JADE1-mediated CCND1/CDKN1B pathway in HEPM cells.

### Inhibition of *miR-4680-3p* Can Partially Restore the Decreased Cell Proliferation Induced by *at*RA

**Figure 4:**
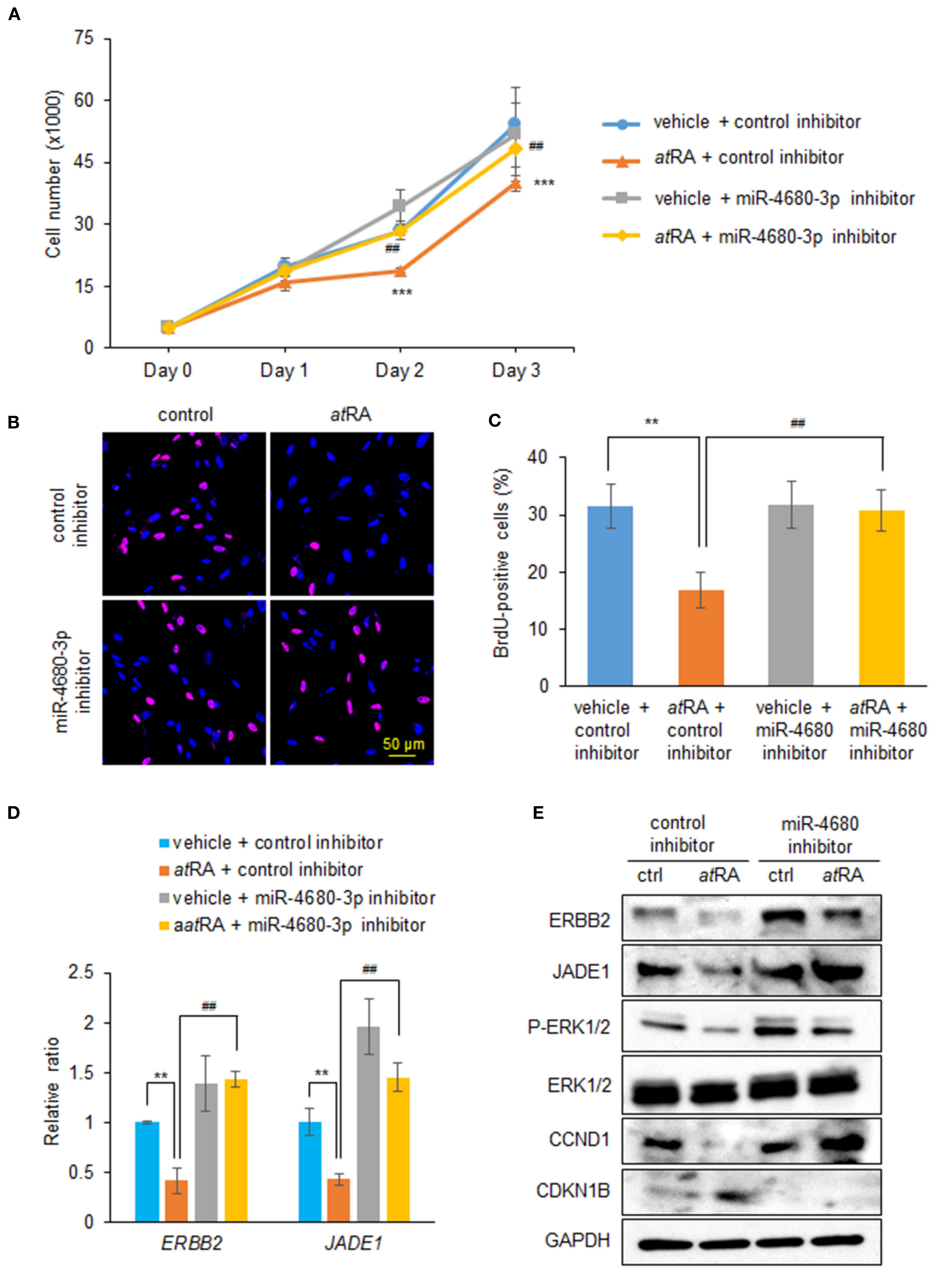
Normalization of *miR-4680-3p* expression restores *at*RA-induced cell proliferation suppression in HEPM cells. **(A)** Cell proliferation assays in HEPM cells treated with 30 μM *at*RA, with/without miR-4680-3p inhibitor, for 24, 48, or 72 h. ***p < 0.001 vs. control inhibitor + vehicle at each indicated day. ^##^*p* < 0.01 vs. control inhibitor + *at*RA at indicated day. **(B)** BrdU staining (red) in HEPM cells after treatment with 30 μM *at*RA, with/without miR-4680-3p inhibitor, for 72 h. Nuclei were counterstained with DAPI (blue). **(C)** Graph shows the quantification of BrdU-positive cells. **p < 0.01 vs. control inhibitor + vehicle. ^##^*p* < 0.01 vs. control inhibitor + *at*RA. **(D)** Quantitative RT-PCR for the indicated genes after treatment with *at*RA, with/without miR-4680-3p inhibitor, for 24 h in HEPM cells. **p < 0.01 vs. control inhibitor + vehicle. ^##^*p* < 0.01 vs. control inhibitor + *at*RA. **(E)** Immunoblotting of ERBB2, JADE1, phosphorylated ERK1/2 (P-ERK1/2), ERK1/2, CCND1, CDKN1B, and GAPDH in HEPM cells treated with 30 μM *at*RA, with/without miR-4680-3p inhibitor, for 72 h. Representative images from two independent experiments are shown.

To examine whether normalization of upregulated *miR-4680-3p* can restore decreased cell proliferation under *at*RA treatment conditions, we treated HEPM cells with a miR-4680-3p inhibitor, with or without *at*RA treatment, and found that a miR-4680-3p inhibitor could partially normalize the reduced cell proliferation (Figures 4A–C). As expected, suppression of *ERBB2* and *JADE1* was normalized by the miR-4680-3p inhibitor under *at*RA treatment conditions (Figures 4D,E). In addition, we confirmed that phosphorylation of ERK1/2 and expression of CCND1 and CDKN1B were normalized with miR-4680-3p inhibitor (Figure 4E). Taken together, our results indicate that *at*RA inhibits cell proliferation through *miR-4680-3p* expression in HEPM cells.

## Discussion

Excessive intake of *at*RA, a teratogenic reagent, induces cleft palate in humans and mice. Previous studies indicate that *at*RA inhibits cell proliferation and induces apoptosis in HEPM cells and mouse embryonic palatal mesenchymal (MEPM) cells (Yu et al., 2005; Dong et al., 2017). Since miRs are postulated to be essential in various biological processes, such as cell proliferation and apoptosis (Chen et al., 2004; Buser and Pohl, 2015; Shirjang et al., 2019; Akkoc and Gozuacik, 2020), we hypothesized that excessive *at*RA induces expression of miRs, which suppress genes crucial for palate development, leading to decreased cell proliferation. Our previous studies show that overexpression of *miR-140-5p, miR-133b, miR-374a-5p, miR-381-3p*, and *miR-4680-3p* suppresses cell proliferation through downregulation of genes related to cleft palate in HEPM cells (Li et al., 2019; Suzuki et al., 2019). Among them, we found that *at*RA specifically induced *miR-4680-3p* expression, which in turn suppressed expression of *ERBB2* and *JADE1*. While expression of some predicted genes targeted by *miR-4680-3p* was not changed by *miR-4680-3p* overexpression in HEPM cells, these genes may be regulated by a combination of other miRs or through feedback loops reported in several cancer cell lines and in C2C12 cells, an immortalized mouse myoblast cell line (Hou et al., 2019; Liu et al., 2019; Quan et al., 2019).

ERBB2 is a member of the ERBB receptor tyrosine kinase family including epidermal growth factor receptor (EGFR) (Yarden and Shilo, 2007). The binding of ligands to receptors induces the homo- or hetero-dimerization of receptors and activates the kinase domain that induces downstream signaling cascades such as MAPK/ERK and PI3K/AKT/mTOR pathways, known to be crucial for cell proliferation, migration, and differentiation (Avraham and Yarden, 2011; Arteaga and Engelman, 2014). Our previous bioinformatic study suggests that ERBB signaling pathway may play a substantial role in palate formation (Yan et al., 2020). In fact, the activation of the ERBB2 pathway upregulates CCND1 expression, decreases CDKN1B stability (Lee et al., 2000; Yang et al., 2000; Lenferink et al., 2001), and promotes cell proliferation and angiogenesis in tumors (Le et al., 2005). In addition, inhibition of the ERBB2 pathway with a neutralizing antibody or small-molecule inhibitor for ERBB2 normalizes CDKN1B expression, leading to cell cycle arrest in human breast cancer cells (Le et al., 2003). *at*RA inhibits cell proliferation through downregulation of CCND1 and upregulation of CDKN1B in HEPM cells (Yu et al., 2005; Dong et al., 2017). Our results show that *at*RA inhibits cell proliferation through upregulated *miR-4680-3p* expression, which suppresses *ERBB2* expression and its downstream ERK1/2 signaling pathway in HEPM cells. Taken together, our findings for the regulation of miRs by *at*RA shed light on the link between environmental and genetic factors, and explain how cell proliferation is inhibited by *at*RA.

JADE1 (a.k.a. PHF17), a transcription factor, contains two variants: JADE1-L (a long form with 842 amino acids) and JADE1-S (a short form without a C-terminal fragment of 333 amino acids) (Borgal et al., 2014; Siriwardana et al., 2015). Although the role of JADE1 remains elusive, the protein exhibits histone acetyltransferase (HAT) activity and acts as a co-factor of the HBO1 complex in histone H4 acetylation during gene regulation, which plays crucial roles in cell cycle regulation (Panchenko et al., 2004; Foy et al., 2008; Panchenko, 2016; Han et al., 2018). The suppression of *JADE1* (both JADE1-L and JADE1-S) by siRNA knockdown results in suppression of DNA synthesis in cultured epithelial cell lines and primary fibroblasts (Havasi et al., 2013). Our results indicate that *at*RA treatment downregulates JADE1 expression, leading to decreased cell proliferation in HEPM cells through downregulated CDKN1 expression and upregulated CDKN1B expression. In future studies, we will characterize JADE1, namely whether and how *JADE1* expression is regulated through the ERK1/2 pathway, and whether and how JADE1 regulates expression of CCND1 and CDKN1B. With those data in hand, we will then be able to conclude that *JADE1* expression is directly regulated by miR-4680-3p, or regulated through ERBB2–mediated ERK1/2 signaling. In case treatment with both miR-4680-3p mimic and ERK1/2 inhibitor induces additional inhibition of cell proliferation, as well as additional suppression of CCND1 expression, *JADE1* expression may be regulated by *miR-4680-3p* through the ERK1/2 pathway.

In summary, our findings and those of others suggest that excessive intake of retinoic acid during pregnancy leads to reduced cell proliferation through dysregulation of *CCND1* and *CDKN1B* mediated by the *miR-4680-3p*–ERBB2–ERK1/2–CCND1/CDKN1B cascade in palatal mesenchymal cells, ultimately leading to cleft palate.

## Data Availability Statement

The original contributions presented in the study are included in the article/Supplementary Material, further inquiries can be directed to the corresponding author/s.

## Author Contributions

HY, SR, and JS performed the experiments. HY, AS, and JI wrote the article. All authors reviewed the results and approved the final version of the article.

## Conflict of Interest

The authors declare that the research was conducted in the absence of any commercial or financial relationships that could be construed as a potential conflict of interest.

## Supplementary Material

Supplementary Figure 1 *at*RA does not induces apoptosis in HEPM cells. **(A)** TUNEL staining (red) in HEPM cells after treatment with 30 μM *at*RA for 72 h. Nuclei were counterstained with DAPI (blue). Scale bar, 50 μm. **(B)** Graph shows the quantification of TUNEL-positive cells. N.D., Not Detected. **(C)** Immunoblotting of cleaved caspase-3 and GAPDH in HEPM cells treated with 30 μM *at*RA for 72 h. ctl, Control; P.C., Positive Control (mouse small intestine). Representative images from two independent experiments are shown.
