# Supplementary figures and images for: Excessive All-Trans Retinoic Acid Inhibits Cell Proliferation Through Upregulated MicroRNA-4680-3p in Cultured Human Palate Cells
Source: Front Cell Dev Biol. 2021 Jan 28;9:618876. doi: 10.3389/fcell.2021.618876 (PMC7876327; doi:10.3389/fcell.2021.618876)

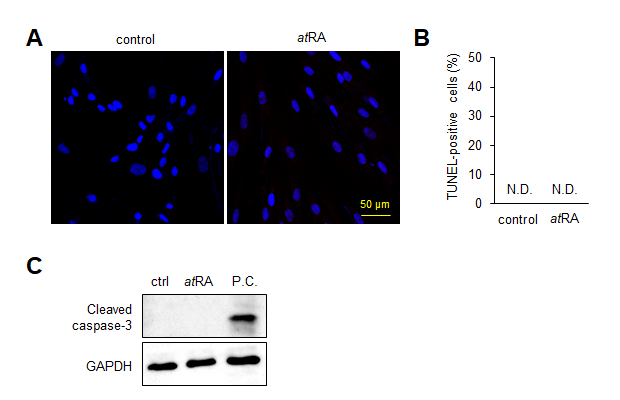

Supplement: Supplementary Figure 1 — atRA does not induces apoptosis in HEPM cells. (A) TUNEL staining (red) in HEPM cells after treatment with 30 μM atRA for 72 h. Nuclei were counterstained with DAPI (blue). Scale bar, 50 μm. (B) Graph shows the quantification of TUNEL-positive cells. N.D., Not Detected. (C) Immunoblotting of cleaved caspase-3 and GAPDH in HEPM cells treated with 30 μM atRA for 72 h. ctl, Control; P.C., Positive Control (mouse small intestine). Representative images from two independent experiments are shown. [file Image_1.TIF]

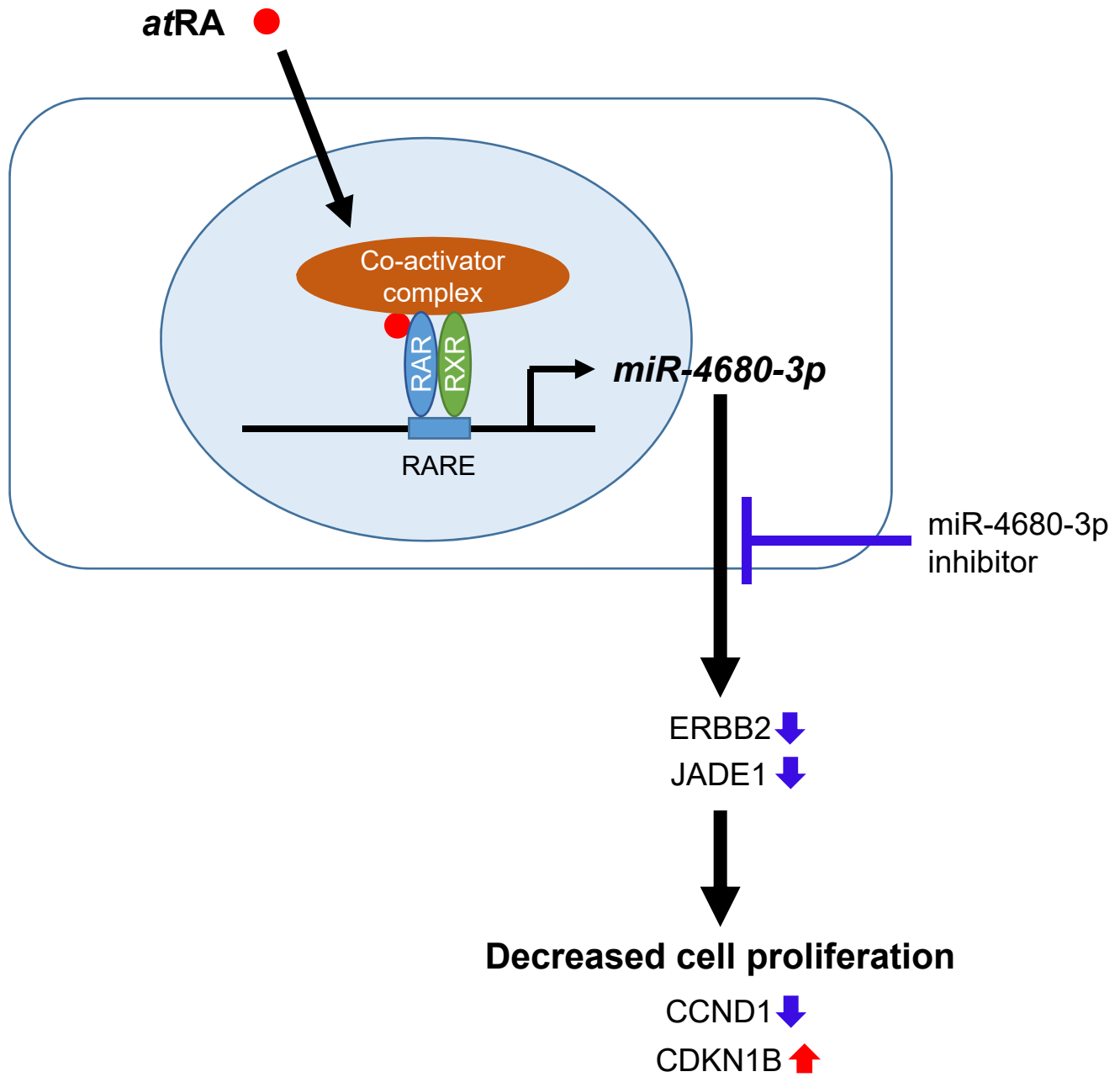

Supplement: Supplementary file 2 [file Data_Sheet_1.PDF]
